# Supplementary material for: Antibiotic Cocktail Effects on Intestinal Microbial Community, Barrier Function, and Immune Function in Early Broiler Chickens
Source: Antibiotics (Basel). 2024 Apr 30;13(5):413. doi: 10.3390/antibiotics13050413 (PMC11117290; doi:10.3390/antibiotics13050413)
Supplement: Supplementary file 1 [file antibiotics-13-00413-s001.zip › antibiotics-2954798-supplementary.pdf]

Supplementary materials

Table S1. Composition and Nutrient levels of the experimental basal diet.

Ingredients	Percentage (%)	Calculated Nutrient levels[3]	
Corn (CP 8.0%)	55.20	Metabolizable energy (Mcal/kg)	3.02
Soybean meal (CP 46.0%)	34.30	Crude protein (%)	20.28
Wheat powder (CP 13.8%)	3.02	Total Calcium (%)	0.95
Soybean oil	3.95	Total phosphorus (%)	0.68
DL-Methionine, 98%	0.20	Lysine (%)	1.22
L-Lysine sulfate, 78%	0.20	Methionine (%)	0.50
Limestone	1.18		
Calcium hydrogen phosphate	1.20		
Sodium chloride	0.30		
DL-Methionine, 98%	0.20		
Vitamin premix[2]	0.03		
Mineral premix[1]	0.15		
Phytase	0.02		
Antioxidants	0.05		
Total	100		

[1]Mineral premix provided per kilogram of complete diet: iron, 80 mg; copper, 8 mg; manganese, 100 mg; zinc, 80 mg; iodine, 0.35 mg; selenium, 0.15 mg. [2]Vitamin premix provided per kilogram of complete diet: vitamin A (retinyl acetate), 12,500 IU; vitamin D3 (cholecalciferol), 2500 IU; vitamin E (DL-a-tocopherol acetate), 30 IU; vitamin K3 (menadione sodium bisulfate), 2.65 mg; vitamin B12 (cyanocobalamin), 0.025 mg; biotin, 0.30 mg; folic acid, 1.25 mg; nicotinic acid, 50 mg; D-pantothenic acid, 12 mg; pyridoxine hydrochloride, 6.0 mg; riboflavin, 6.5 mg; thiamine mononitrate, 3.0 mg.[3]Calculated value based on analysis of the experimental diets.

Table S2. Sequences of the oligonucleotide primers used for quantitative real-time PCR.

Gene Name	Primer Sequence (5' to 3')	GenBank Accession
β-actin	F: CAACACAGTGCTGTCTGGTGGTAC<br>R: CTCCTGCTTGCTGATCCACATCTG	L08165
ZO-1	F: CTTCAGGTGTTTCTCTTCCTCCTC<br>R: CTGTGGTTTCATGGCTGGATC	XM_015278981.2
Occludin	F: ACGGCAGCACCTACCTCAA<br>R: GGGCGAAGAAGCAGATGAG	NM_205128.1
Claudin-1	F: CATACTCCTGGGTCTGGTTGGT<br>R: GACAGCCATCCGCATCTTCT	NM_001013611.2
FABP-2	F: TGGAAGCAATGGGCGTGAAT<br>RTGTCGATGGTACGGAAGTTGC	NM_001007923.1
Mucin-2	F: TTCATGATGCCTGCTCTTGTG<br>R: CCTGAGCCTTGGTACATTCTTGT	XM-040701667.1
IL-1β	F: ACTGGGCATCAAGGGCTA<br>R: GGTAGAAGATGAAGCGGGTC	XM_015297469.1
IL-6	F: CGCCCAGAAATCCCTCCTC<br>R: AGGCACTGAAACTCCTGGTC	XM_015281283.1
TNF-α	F: GAGGGTTGACTTGGCTGTC<br>R: AAGCAACAACCAGCTATGCAC	NM204267.2
IFN- γ	F: AAAGCCGCACATCAAACA<br>R: GCCATCAGGAAGGTTGTTTTTC	CA NM205149.1
IL-8	F: GGCTTGCTAGGGGAAATGA<br>R: AGCTGACTCTGACTAGGAAACTGT	AJ009800

Primers were synthesized by Sangon Biotech Co., Ltd. (Shanghai).

Table S3. The relative abundance of bacterial communities at the phylum and genus level in the cecum and ileum of broilers after 14 days of antibiotic cocktail (ABX) treatment.

Cecal bacterial communities	Antibiotic cocktail treatments			analysis	
	CC14	AC14	BC14	SEM	*p* value
**Phylum**					
Firmicutes	97.94^{a}	89.76^{a}	54.26^{b}	7.29	0.025
Bacteroidetes	0.80	3.60	4.10	1.02	0.370
Actinobacteria	0.67	0.72	1.13	0.31	0.650
Proteobacteria	1.40	5.71	3.47	1.29	0.395
Cyanobacteria	0.215	0.15	0.37	0.09	0.874
**Genus**					
Enterococcus	0.21^{b}	0.39^{b}	59.42^{a}	8.56	0.001
Unclassified_Lachnospiraceae	19.08^{a}	16.29^{a}	4.81^{b}	2.24	0.012
Unclassified_Oscillospiraceae	8.93^{a}	7.49^{a}	1.58^{b}	0.84	0.000
Ruminococcus_torques_group	6.09^{ab}	8.88^{a}	2.67^{b}	1.04	0.039
Unclassified_Ruminococcaceae	6.65^{a}	6.78^{a}	1.43^{b}	0.85	0.005
**Ileal bacterial communities**	**Antibiotic Cocktail Treatments**			**Analysis**	
	**CI14**	**AI14**	**BI14**	**SEM**	***p* value**
**Phylum**					
Firmicutes	96.73^{a}	95.73^{a}	81.17^{b}	2.42	0.008
Bacteroidetes	1.65^{b}	1.03^{b}	3.68a	0.580	0.005
Actinobacteria	0.21	0.53	1.02	0.1593	1.040
Proteobacteria	1.03	1.03	33.94	0.517	0.420
Cyanobacteria	0.74^{b}	0.97^{b}	6.98^{a}	0.098	0.004
**Genus**					
Enterococcus	0.59^{b}	1.289^{b}	63.40^{a}	7.80	0.001
Bacillus	5.29^{ab}	13.71^{a}	2.08^{b}	2.06	0.048
Unclassified_Lachnospiraceae	5.09	1.69	1.03	0.28	0.250
Unclassified_Ruminococcaceae	2.22	0.832	0.51	0.47	0.295
Lactobacillus	56.16^{a}	49.80^{a}	5.48^{b}	6.10	0.001

SEM, standard error of the mean; CC (cecum) and CI (ileum) control groups basal feed along boiled water only, while AC&BC (cecum) and AI &BI (ileum) were fed basal diet with (ABX for 7 days) and (ABX for 14 days) in the drinking water the rate of 2.5g/L, respectively. ^{a,b,c}Values with different superscripts differ significantly ($p < 0.05$).

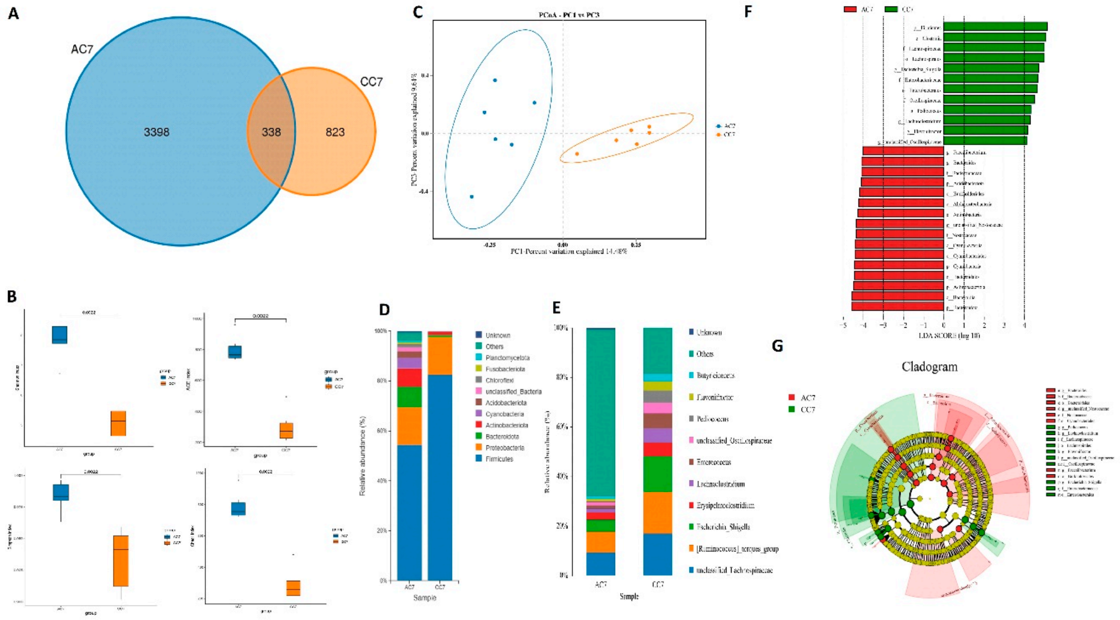

Figure S1. Antibiotic cocktail effecting the composition and function of the cecal microbiota after 7 days of sampling. **(A)** Among the total 4221 operational taxonomic units (OTUs), antibiotic-treated AC7 contains 3398 OTU while CC7 has 823 OUT, though both commonly share 338 OUT. **(B)** The alpha diversity as measured by the CE index (i. ACE index, ii. Chao index, iii. Shannon, iv. Simpson) shows a significantly higher level in AC7 group as compared to CC7. **(C)** The beta diversity using principal component analysis (PCoA), demonstrates significantly different microbial communities among both groups (AC7 and BC7). **(D)** The microbial relative abundance at phylum level shows Firmicutes significantly higher (≅ 85%) in CC7 and relatively lower (65%) in AC7. The relative abundance of Proteobacteria is significantly lower, but Bacteroides and *Actinobacteria* are significantly higher in AC7 group as compared to CC7. **(E)** The microbial relative abundance at genus level, reveals unclassified Lachnospiraceae bacteria are more abundant in control group (CC7) as compared to antibiotic cocktail-treated group (AC7). **(F)** LEfSe analysis of intestinal microbiota composition after antibiotic cocktail treatment. There are higher levels of Firmicutes and Bacteroides in CC7 and AC7, respectively, at the LDA score (log 10). (**G)** Cladogram exhibiting differential bacteria in the control group with a significant difference in the antibiotic group.

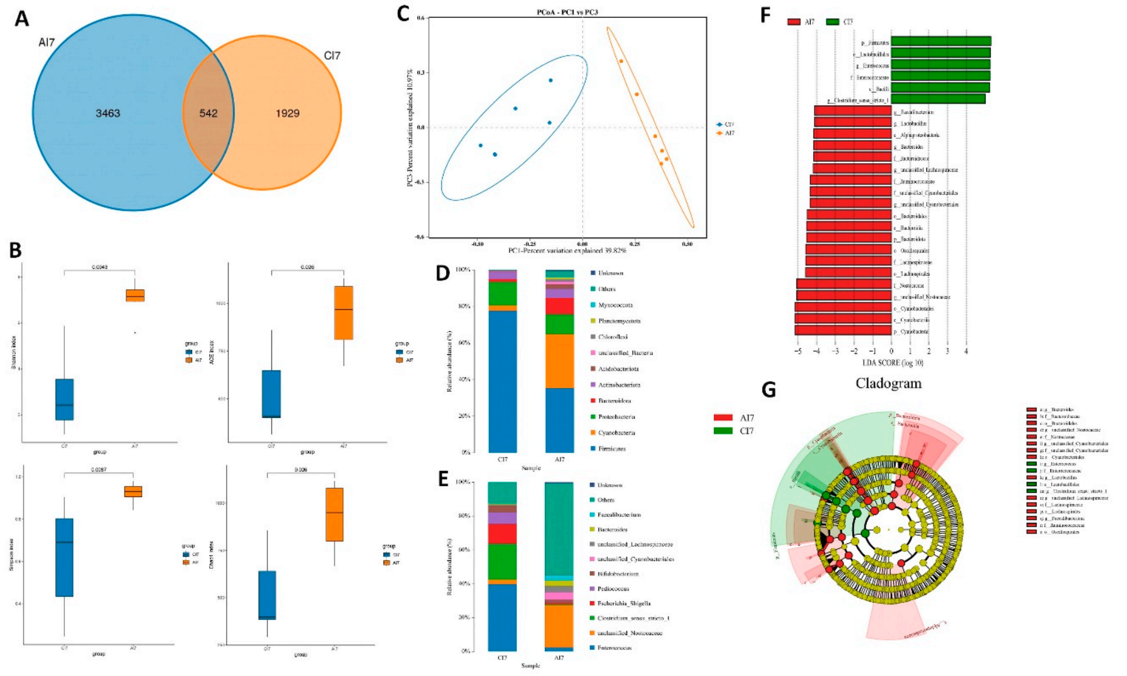

Figure S2. Antibiotic cocktail affecting the composition and function of the ileal microbiota after 7 days of sampling. **(A)** A total of 5392 operational taxonomic units (OTUs) were obtained from both groups, CI7 and AI7, in which the AI7 group has 3463 unique OTUs and CI7 group has 1929 unique OTUs, and both groups share 542 common OTUs. **(B)** Alpha diversity from all CE indexes (i. ACE index, ii. Chao index, iii. Shannon, iv. Simpson) is significantly higher in the AI7 group as compared to CI7. **(C)** Beta diversity, using principal component analysis (PCoA), shows significantly different microbial communities among both groups in the ileum. **(D)** The ileal microbial relative abundance at phylum, mainly composed of Firmicutes, Cyanobacteria, Proteobacteria, and Bacteriota, has no significant difference in the dominant phyla between the two groups ($p > 0.05$). **(E)** The relative abundance at the genus level demonstrates the abundance of Firmicutes from dominant bacteria is significantly lower ($p > 0.05$) in group AI7 as compared to CI7. **(F)** LEfSE analysis of ileal microbiota composition shows that the dominant bacteria, Firmicutes, Lactobacillus, *Enterococcus*, and Enterococci, are more enriched in CI7 as compared to A17. While Nostocaceae, *F*_Nostococci, *D*_Cynobacteria, and *P*_Cynobacteria are dominant in AI7 group as compared to C17 group. **(G)** Cladogram showing variance in bacteria in the control group with a substantial difference in the antibiotic group.
